# Effects of plasma-activated Ringer’s lactate solution on cancer cells: evaluation of genotoxicity

**DOI:** 10.1186/s41021-023-00260-x

**Published:** 2023-01-13

**Authors:** Yang Liu, Yoshimichi Nakatsu, Hiromasa Tanaka, Kazunori Koga, Kenji Ishikawa, Masaharu Shiratani, Masaru Hori

**Affiliations:** 1 Graduate School of Engineering, Nagoya University, Furo-cho, Chikusa-ku, Nagoya, 464-8603 Japan; 2 Graduate School of Medical Sciences, Kyushu University, Fukuoka, Japan; 3 Present address: Cancer Genetics Laboratory, Clinical Research Institute, NHO Kyushu Cancer Center, Fukuoka, Japan; 4 Center for Low-temperature Plasma Sciences, Nagoya University, Nagoya, Japan; 5 Graduate School of Information Science and Electrical Engineering, Kyushu University Fukuoka, Fukuoka, Japan

**Keywords:** PAL, Genotoxicity, Cytotoxicity

## Abstract

**Background:**

Non-thermal atmospheric pressure plasma technologies form the core of many scientific advances, including in the electronic, industrial, and biotechnological fields. The use of plasma as a cancer therapy has recently attracted significant attention due to its cancer cell killing activity. Plasma-activated Ringer’s lactate solution (PAL) exhibits such activity. In addition to ROS, PAL contains active compounds or species that cause cancer cell death, but the potential mutagenic risks of PAL have not been studied.

**Results:**

PAL has a low pH value and a high concentration of H_2_O_2_. H_2_O_2_ was removed from PAL using catalase and catalase-treated PAL with a pH of 5.9 retained a killing effect on HeLa cells whereas this effect was not observed if the PAL was adjusted to pH 7.2. Catalase-treated PAL at pH 5.9 had no significant effect on mutation frequency, the expression of γH2AX, or G2 arrest in HeLa cells.

**Conclusion:**

PAL contains one or more active compounds or species in addition to H_2_O_2_ that have a killing effect on HeLa cells. The compound(s) is active at lower pH conditions and apparently exhibits no genotoxicity. This study suggested that identification of the active compound(s) in PAL could lead to the development of novel anticancer drugs for future cancer therapy.

## Introduction

Plasma, the fourth state of matter, differs from solid, liquid and gas phases. It is estimated that more than 99% of the matter in the visible universe exists in the plasma state. The typical examples of plasma in nature are auroras and lightning. Plasma is composed of electrons, various ions, ultraviolet/vacuum ultraviolet (UV/VUV) radiation, photons, and reactive oxygen/nitrogen species (ROS/RNS), including ozone, hydrogen peroxide, singlet oxygen, superoxide, hydroxyl radical and nitric oxide. The thermal equilibrium states of plasma are mainly equilibrium plasma and non-equilibrium plasma. High densities of reactive species are generated in non-equilibrium plasma near room temperature due to selective heating of electrons by electric fields, while ions and neutral species remain unheated. Plasma technologies are at the core of the electronic industrial and biotechnological fields, including surface modification (hydrophilization, hydrophobicity, water repellency) [1, 2], etching (SiO_2_, Si, organic films) [3, 4], waste decomposition treatment [5], cleaning (substrate, chamber, etc.) [6, 7], nanoscience [8], biotechnological materials [9, 10], sterilization [11, 12], light sources (VUV, EUV, etc.) [13] and agriculture [14–16]. Plasma is also used in elemental analysis and environmental monitoring [17]. Non-equilibrium atmospheric pressure plasma (NEAPP) has recently been used in the biomedical field [18, 19] in direct or indirect way. Direct treatment consists of applying NEAPP exposure directly on in vitro cells or in vivo system, and NEAPP can also be applied on cells or tumors indirectly, by using plasma activated solution. Plasma holds potential as a cancer therapy because it induces cell death (apoptosis) specifically in cancer cells.

Tanaka et al. reported that plasma-irradiated medium (PAM), a kind of indirect plasma treatment, induces apoptosis in human brain tumor cells (U251SP) but not in normal human astrocytes (ACBRI-371), suggesting the selective killing effect of PAM on cancer cells [20]. Similar effects have been observed using various types of human cancer cells, including gastric cancer [21], liver and breast cancer [22], colorectal cancer [23], ovarian clear cell adenocarcinoma [24], and small cell lung carcinoma [25]. Furthermore, PAM induces apoptosis in ovarian cancer cell lines resistant to anticancer drugs such as paclitaxel and cisplatin. These results suggest that PAM treatment may be effective for cancer therapy, including for chemotherapeutic agent-resistant cancer.

Culture medium is complex and contains inorganic salts such as sodium chloride, amino acids, vitamins, and glucose, and thus the reactions occurring in culture medium upon irradiation with plasma are complicated, preventing the comprehensive analysis of reaction mechanisms and the identification of any new active compounds. In contrast, Ringer’s lactate solution (lactec) can be used in place of culture medium for many practical applications and is composed of only four compounds: sodium chloride, potassium chloride, calcium chloride, and sodium lactate. Lactec irradiated with plasma is called plasma-activated lactec (PAL) [26–29]. Tanaka et al. reported the selective killing of brain tumor cells upon the addition of PAL, similar to the effect of direct irradiation by plasma on cancer cells. Both ROS and plasma-activated sodium lactate exhibit killing effects on cancer cells [26]. Furthermore, the intraperitoneal administration of PAL into mice reportedly reduces the tumor volume of transplanted cervical cancer cells without affecting the weight of the mice [27].

Many of the radicals generated during plasma discharge can contribute to complex reactions and the formation of other chemically active species in PAL that potentially have killing effects on cancer cells. The mechanism of PAL-induced cell death appears to involve metabolomic changes but not oxidative stress [30]. The regulation of apoptosis is important in cancer biology because many cancer cells have defective apoptosis processes [31]. DNA damage can cause cell cycle arrest, which leads to apoptosis in cancer cells [32]. PAL contains ROS and active compounds or species that cause cancer cell death, but the potential mutagenic risks of PAL are currently not understood. Here, we investigated the cytotoxic and genotoxic activity of PAL using the HeLa human cervical carcinoma cell line.

## Materials and methods

### Cells and culture

HeLa cells (human cervical carcinoma cell line obtained from Prof. M. Sekiguchi, Kyushu University) were cultured in Dulbecco’s Modified Eagle Medium (D-MEM) (Gibco, Thermo Fisher Scientific, Waltham, MA, USA) supplemented with 10% fetal bovine serum (FBS) (Sigma-Aldrich, St. Louis, MO, USA), 100 units/mL penicillin and 100 μg/mL streptomycin (Gibco, Thermo Fisher Scientific) at 37 °C in air containing 5% CO_2_ [33].

### Preparation of PAL

Lactec (Ringer’s lactate solution, Na^+^ 130 mEq/L, K^+^ 4 mEq/L, Ca^2+^ 3 mEq/L, Cl^−^ 109 mEq/L, L-lactate 28 mEq/L, pH 6.0 ~ 7.5, Otsuka Pharmaceutical Factory, Inc., Osaka, Japan; 3 mL) in a 35-mm tissue culture dish was irradiated using NEAPP [argon gas flow rate: 2.0 slm (L/min.), voltage: 9 kV_p-p_, frequency: 60 Hz; distance between the plasma source and the liquid surface: 6 mm] for 1 to 10 min. After irradiation, the PALs were stored at − 80 °C until use (at most 3 months). The frozen PALs were thawed at 4 °C for 3 h and kept at room temperature (26.3 ± 0.4 °C) for 1 h before use, then filtered through a Millipore Millex-GV 0.22 μm filter (Merck, Darmstadt, Germany). H_2_O_2_ was removed from PAL by adding 100 units/mL of catalase (Cat. from bovine liver, No. 035–12,903, Fujifilm Wako Pure Chemical Corp., Osaka, Japan) and the mixture was incubated for 10 min at room temperature (26.3 ± 0.4 °C). No hydrogen peroxide were detected (less than 0.3 μM) after this treatment.

### Hydrogen peroxide concentration

The concentrations of H_2_O_2_ generated in PAL and diluted PAL were determined with the colorimetric method using an Amplex Red Hydrogen Peroxide Assay kit (Invitrogen, Thermo Fisher Scientific, Waltham, MA, USA) and by using a Digital Packtest hydrogen peroxide (DPM2-H_2_O_2_, Kyoritsu Chemical-check Lab., Corp., Yokohama, Japan) following the manufacturers’ protocols.

### pH adjustment of PALs

The pH values of PAL and diluted PAL were measured with a pH meter (LAQUA, HORIBA, Ltd., Kyoto, Japan) and adjusted by adding 20% (vol/vol) of either 0.1 M phosphate buffer (pH 7.2) or 0.1 M citrate buffer (pH 6.1).

### WST-8 assay for cell viability

The WST-8 assay (Cell Counting Kit-8, Dojindo Laboratories Co. Ltd., Kumamoto, Japan) was performed to determine the viability of the HeLa cells. The cells were harvested using trypsin-EDTA (Gibco, Thermo Fisher Scientific) and then seeded in 96-well plates (Falcon, Corning Inc., Glendale, AZ, USA). The plates were pretreated with 60 μL/well of 40.6 μg/mL collagen type I (Corning Inc.) for 30 min before use. The cells (5000 cells/100 μL) were added to each well, incubated for 24 h at 37 °C in a CO_2_ incubator, then treated with 100 μL of PAL for 120 min. After removing the test solution, the cells were cultured in 100 μL of fresh medium for 24 h, then 10 μL of WST-8 reagent was added to each well. After 2 h incubation at 37 °C in the dark, the absorbance at 450 nm was measured with a microplate reader (Multiskan GO, Thermo Fisher Scientific). Cell viability was calculated as follows: [(absorbance of samples)-(absorbance of background)] / [(absorbance of control) - (absorbance of background)] × 100%, where background represents the mixture of WST-8 reagent and D-MEM without cells.

### Western blot analysis

Cells (2.0 × 10^6^) were directly lysed in 100 μL of 2 × SDS sample buffer [62.5 mM Tris-HCl (pH 6.8), 2% (w/v) SDS, 25% (w/v) glycerol, 0.01% (w/v) bromophenol blue, 5% (v/v) 2-mercaptoethanol] (Bio- Rad, Hercules, CA, USA), incubated at 100 °C for 10 min and then chilled on ice. After sonication for 10 min (30 seconds on, 30 seconds off) the cell lysates (10 μL) were subjected to SDS-polyacrylamide gel electrophoresis and the separated proteins were transferred onto 0.2 μm pore size PVDF membranes (Bio-Rad). The membranes were blocked with 2% skimmed milk (Nacalai Tesque, Kyoto, Japan) in PBS containing 0.1% tween 20 (Nacalai Tesque), then reacted with anti-γH2AX (phosho S139) rabbit monoclonal antibody EP854(2)Y (Abcam, PLS Cambridge, UK) and anti-β-actin mouse monoclonal antibody AC-15 (Sigma-Aldrich). The membranes were further reacted with an appropriately diluted anti-mouse or anti-rabbit IgG conjugated with horseradish peroxidase (HRP) (GE Healthcare, Chicago, IL, USA), followed by reaction with Luminata™ Forte HRP substrate (Merck Millipore, Burlington, MA, USA). Luminescent signals were visualized using a ChemiDoc™ MP system (Bio-Rad).

### Mutation frequency

Cells were cultured in D-MEM containing 10% FBS, 100 units/mL penicillin and 100 μg/mL streptomycin and HAT supplement (0.1 mM hypoxanthine, 0.4 μM aminopterin and 16 μM thymidine) (Gibco, Thermo Fisher Scientific) for 2 weeks to eliminate hypoxanthine-guanine phosphoribosyltransferase (HPRT)-mutated cells from the cell population. The cells were then cultured in the same medium without aminopterin and 1.0 × 10^6^ cells were treated with PAL or H_2_O_2_ for 2 h. The treated cells were cultured in normal medium to about 1 × 10^7^ cells. Exponentially growing cells were divided into five 100-mm dishes at a density of 2 × 10^6^ cells/dish and cultured in medium supplemented with 10 μM 6-thioguanine (Fujifilm Wako Pure Chemical Corporation) for 10 days to form colonies. To determine plating efficiency, 400 cells were inoculated onto a 100-mm dish, the formed colonies were fixed with 3.7% formaldehyde (Nacalai Tesque) in PBS, stained with 0.1% crystal violet (Fujifilm Wako Pure Chemical Corporation) and counted. Mutation frequency was calculated from the number of resistant colonies and the plating efficiency.

### Cell cycle analysis

Cells were harvested with trypsin/EDTA and washed with PBS. Cell suspensions were passed through a cell strainer (Falcon, Corning Inc., Glendale, AZ, USA) and centrifuged. After removing the supernatants, cell pellets (1.0 × 10^6^ cells) were suspended in 50 μL of PBS and the cells were fixed with 70% ethanol at − 30 °C. The fixed cells were passed through a cell strainer and centrifuged at 300×*g* for 5 min. After washing with PBS, the cells were suspended in 200 μL of Muse cell cycle reagent (Merck Millipore) and incubated at room temperature for 30 min in the dark. Cell cycle analyses were carried out following the recommended Muse Cell Analyzer (Merck Millipore) protocol.

## Results

### pH and hydrogen peroxide in PAL

**Fig. 1:**
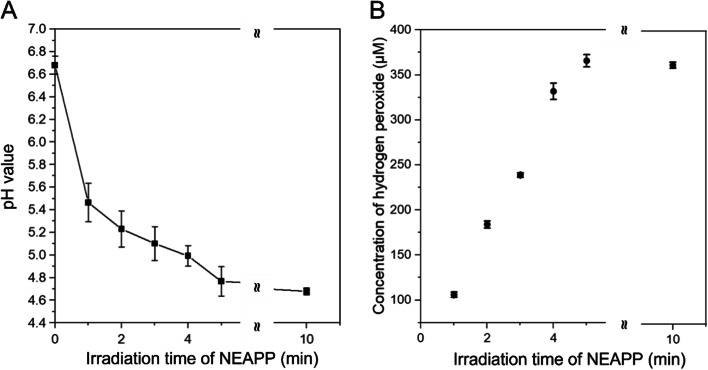
**A** The pH value of PALs (after thawing at 4 °C for 3 h) and **B** the concentration of H_2_O_2_ generated in PALs with irradiation time. Irradiation time 0 represented untreated lactec

The pH value and concentration of hydrogen peroxide (H_2_O_2_) in PAL samples generated by NEAPP irradiation for 1, 2, 3, 4, 5, or 10 min were measured after thawing the samples at 4 °C for 3 h. Untreated lactec had a pH of 6.7 ± 0.1. The pH values of the PALs decreased with increasing plasma exposure time, as shown in Fig. 1A. Longer NEAPP irradiation introduces more hydrogen ions (H^+^) into lactec from the air, and the dehydrogenation of lactate [34] also results in the reduction of pH. The amount of H_2_O_2_ generated increased with increased irradiation time (Fig. 1B) and plateaued around 360 μM in the 5-min irradiation PAL sample.

### Effect of pH on cell viability

Many activated organic species in addition to ROS are generated in the interaction of plasma and sodium lactate [26, 27, 30] and may induce the apoptosis of cancer cells. However, lactec is not a buffered solution, which prevents the effective regulation of pH during cancer treatment.

**Fig. 2:**
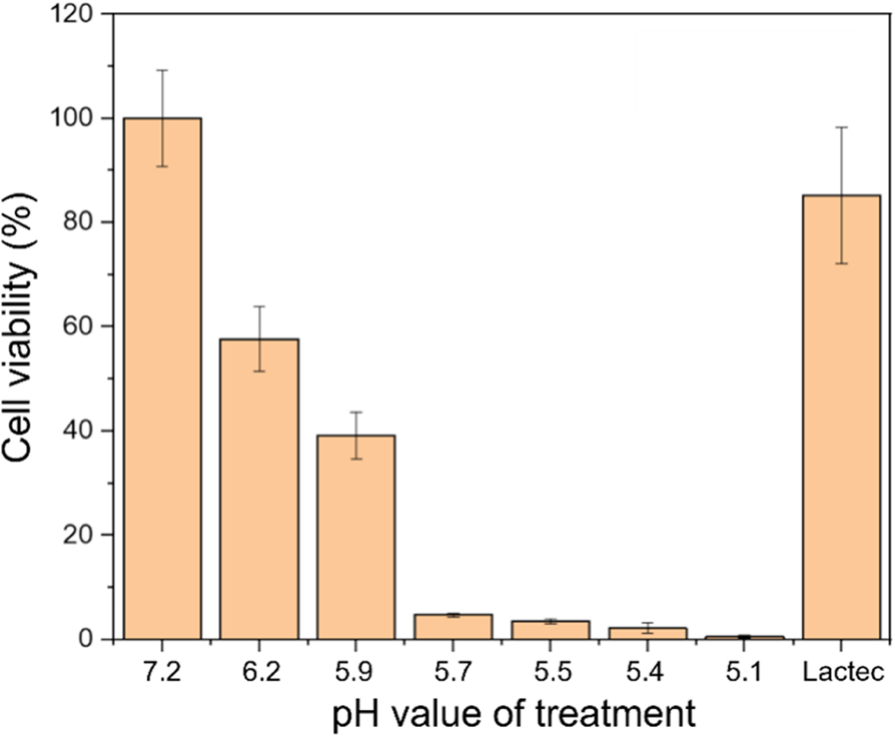
Cell viability of HeLa cells treated with buffered lactec with different pH values. Lactec solutions were pre-treated with phosphate buffer 7.2 or citrate buffer to adjust the pH value as 5.1 to 6.2. HeLa cells were treated with buffered and untreated-lactec solution for 2 h. The cell survival test was performed 24 h after changing the medium

To evaluate the effect of low pH on HeLa cells, cells were treated with phosphate-buffered (pH 7.2), citrate-buffered (pH 6.2 ~ 5.1) or unbuffered lactec (pH 6.7) for 2 h, then cultured in normal medium for 24 h and the cell viabilities were determined. Treatment with low pH lactec profoundly affects the viability of HeLa cells (Fig. 2), with fewer than 5% of the cells surviving when the pH of the solution was below 5.7. The pH of PALs was below 5.6, as shown in Fig. 1A, indicating that we cannot directly use PAL for cell biological assays involving HeLa cells. Therefore, phosphate- or citrate-buffered PAL was used in the following experiments.

### Treatment with pH-adjusted and catalase-treated PALs

**Fig. 3:**
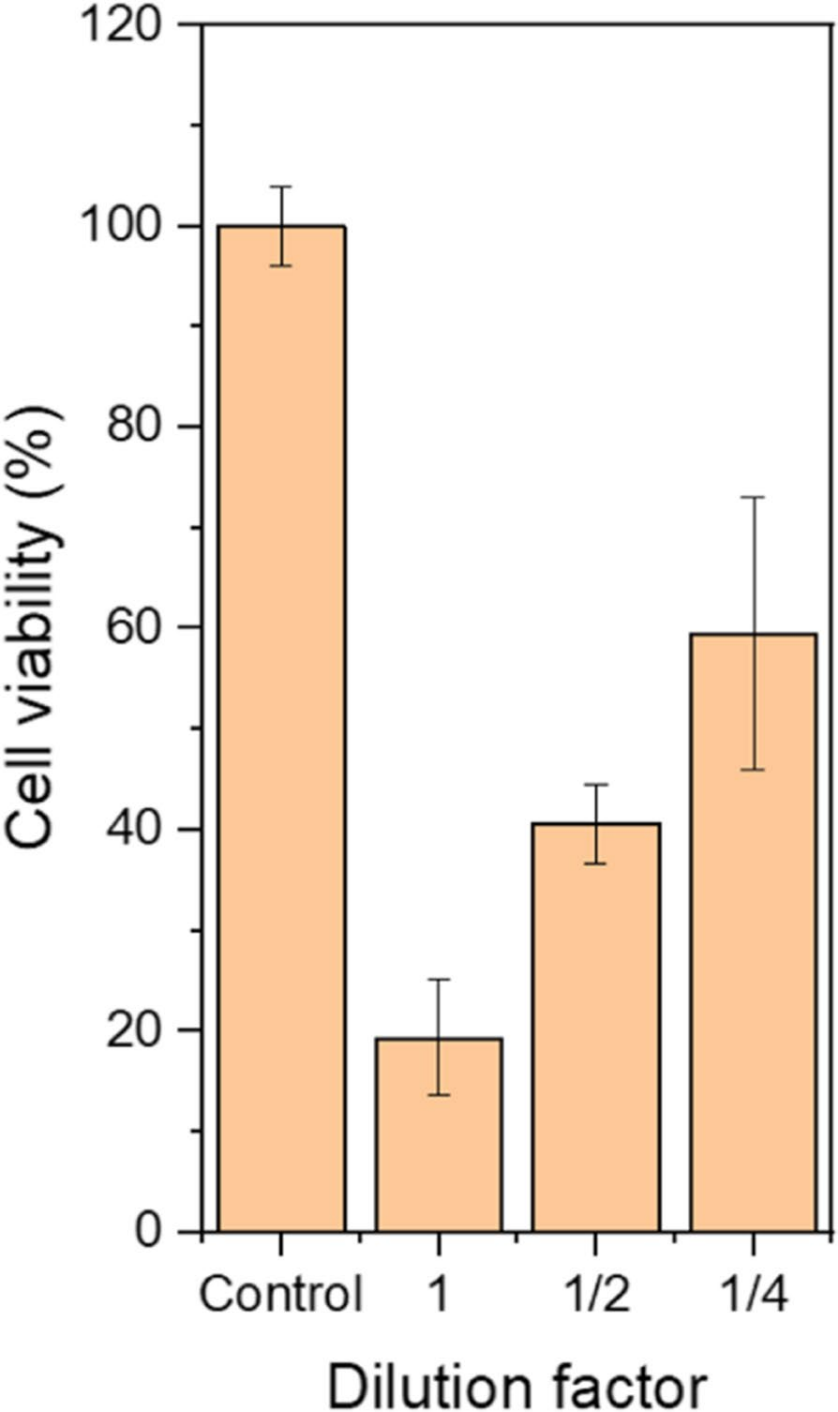
Viability of HeLa cells treated with diluted, catalase-treated and citrate-buffered 10-min PALs. Lactec (control) and PALs were treated with 100 units/mL of catalase and adjusted to pH 6.1 with 20 mM citrate buffer. PALs were 1- to 4-fold diluted with pH-adjusted lactec before used to treat the cells. The viabilities were determined using the WST-8 assay

HeLa cells were used to investigate the killing effects on cancer cells of any active compounds in addition to hydrogen ions and hydrogen peroxide in PAL. Cells were treated with pH-adjusted and catalase-treated PALs. PAL with a pH of 7.2 completely killed the cells whereas HeLa cells treated with catalase-treated PAL with a pH of 7.2 were unaffected. However, a significant dose-dependent killing effect on HeLa cells was observed when the cells were treated with catalase-treated and citrate-buffered 10 min-PAL (PAL irradiated with NEAPP for 10 min) with a pH of 5.9, as shown in Fig. 3. Similar but weaker effects were observed when the cells were subjected to catalase-treated and citrate-buffered 5-min PAL (pH 6.0), with about 50% of the cells surviving following treatment with undiluted PAL. Slightly fewer cells treated with 2-fold-diluted, catalase-treated or citrate-buffered 10-min PALs survived compared to those treated with 5-min PAL (catalase-treated and citrate-buffered), suggesting the presence of active compounds in addition to H^+^ and H_2_O_2_, generated during NEAPP-irradiation in an exposure time-dependent manner.

### Colony-forming ability in low pH conditions

**Fig. 4:**
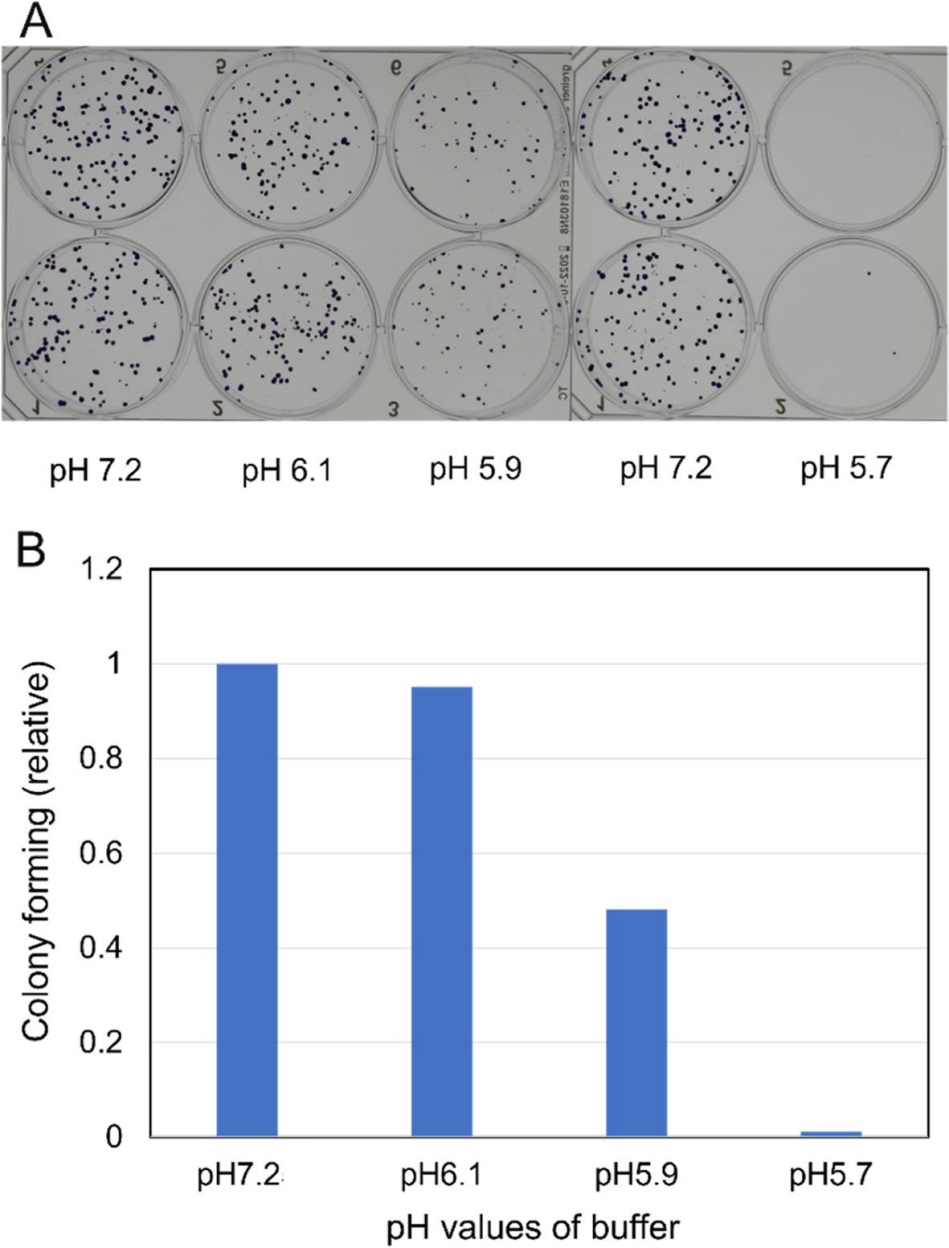
Colony formation by HeLa cells after treatment with lactec at various pH values. **A** The photos show stained colonies formed in the wells of 6-well plates after treatment with 20 mM phosphate-buffered (pH 7.2) or 20 mM citrate-buffered (pH 6.1 ~ 5.7) lactec. **B** The relative colony-forming ability of HeLa cells treated with lactec containing 20 mM buffer with different pH values (pH 7.2: phosphate buffer, 6.1 ~ 5.7: citrate buffer)

The WST-8 assay results suggest that a low pH value for PAL greatly decreases colony formation by HeLa cells. Therefore, we first determined the colony-forming ability of HeLa cells after exposure to low pH conditions prior to determining the mutation frequency at the *HPRT-1* locus using PAL-treated HeLa cells. As shown in Fig. 4A and B, the number of colonies obtained following treatment with citrate-buffered lactec at pH 6.1 is close to that of cells treated with phosphate-buffered lactec at pH 7.2 (control). However, treating cells with citrate-buffered lactec at pH 5.9 decreases the number of colonies by over 50% compared with the control. Cells treated with lactec at pH 5.7 resulted in fewer than 2% of the colonies observed compared with the control. These results indicate that the pH value of PAL impacts colony formation and thus the final pH values of the PALs were adjusted to above 5.9 in subsequent analyses.

**Fig. 5:**
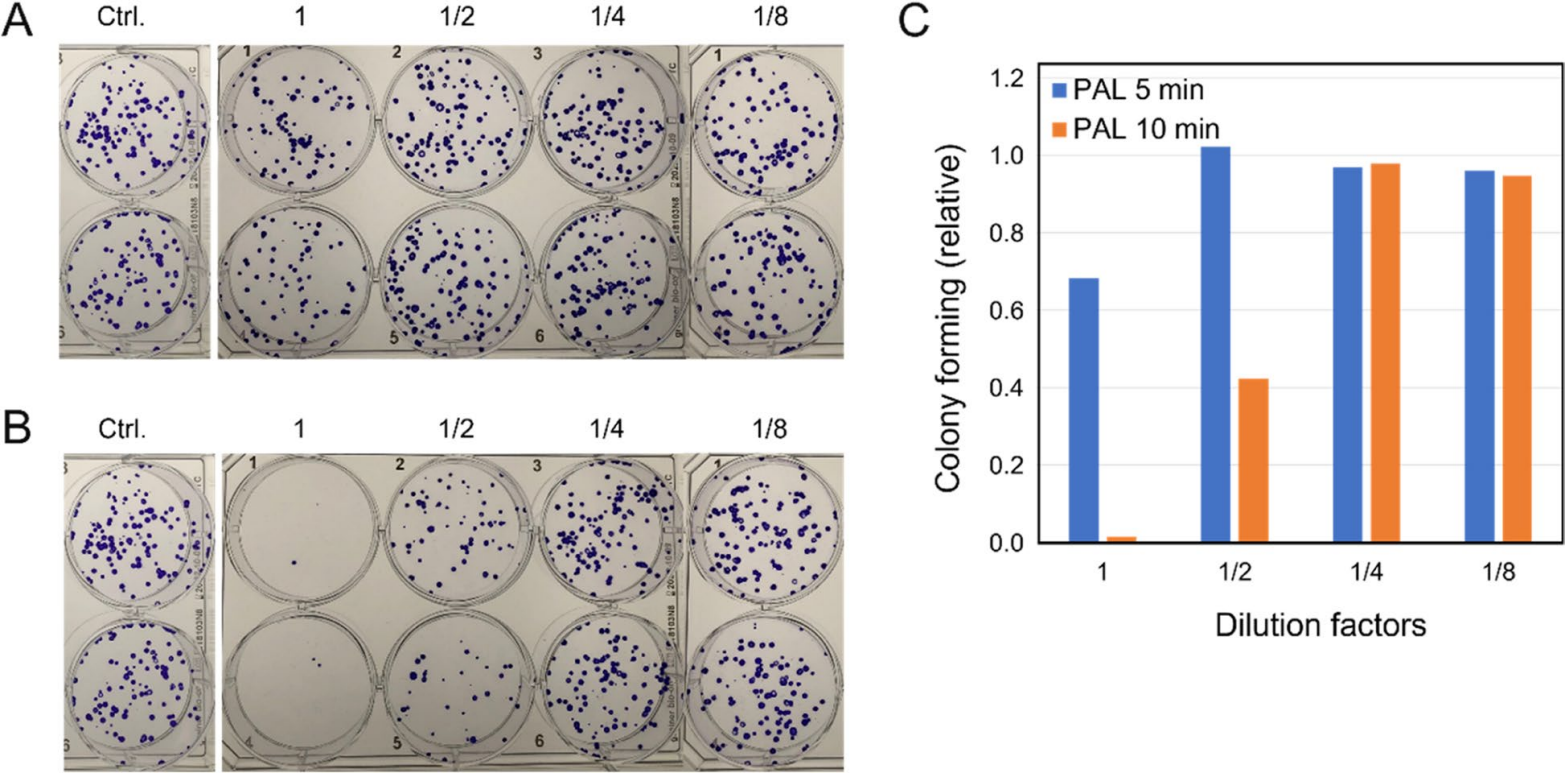
Colony-formation by HeLa cells treated with lactec (Ctrl.) and 1- to 8-fold diluted **A** 5-min PAL or **B** 10-min PAL. The pH of the solutions was adjusted with 20 mM citrate buffer (pH 6.1) and then the PALs were treated with catalase. The photos show colonies in the wells of 6-well plates after fixation and staining. The dilution factors are shown above the panels. **C** Relative colony forming ability of the cells. The number of colonies in the control (treated with buffered lactec) is normalized as “1”

After adjusting the pH values of the PALs with citrate buffer (pH 6.1), HeLa cells were treated with undiluted or 2-fold diluted 5-min and 10-min PALs. The pH values of the citrate buffered 5-min PAL and 10-min PAL were 6.0 and 5.9, and those of the 2-fold diluted PALs were 6.1 and 6.0, respectively. The colony-forming abilities of the cells were determined by inoculating 150 cells/well in 6-well plates (Fig. 5A, B). Relative colony formation is shown as histograms in Fig. 5C. Significant and dose-dependent killing effects were observed, similar to the WST-8 assay results when the cells were treated with catalase-treated and citrate-buffered 10-min PALs, with the catalase-treated and citrate-buffered 5-min PALs having a weaker effect. These results suggest that active compounds or species in addition to H^+^ and H_2_O_2_ are generated in PAL upon NEAPP-irradiation in an exposure time-dependent manner.

### Mutation frequency of PAL-treated HeLa cells

Mutation analyses using the *HPRT1* locus were performed with HeLa cells treated with diluted pH-adjusted 5-min or 10-min PALs with or without catalase treatment, and the mutation frequencies were compared to those of cells cultured in pH-adjusted lactec. The colony-forming ability was determined following each treatment: 16-fold diluted 5-min PAL, 30%; 32-fold diluted 10-min PAL, 50%; 5-min PAL with catalase, 73%; 1.5-fold diluted 10-min PAL with catalase, 35%. Diluted PAL without catalase treatment increased the mutation frequency in HeLa cells: 15.50-fold increase with diluted 5-min PAL and 5.44-fold increase with diluted 10-min PAL. However, catalase-treated PAL had no apparent effect on the mutation frequency of HeLa cells (Table 1). These results indicate that the mutagenicity effect of PAL is mainly caused by H_2_O_2_ and is dependent on the concentration of hydrogen peroxide.

**Table 1:** The mutation frequency of HeLa cells treated with PALs

Treatment ^a), b)^	H_2_O_2_ Concentration (μM)	Mutation Frequency (10^−7^)	Relative Mutation frequency
Lactec	N.D.^c)^	10.4 ± 0.7	1.00
16-fold diluted 5-min PAL	65.3	161.2 ± 9.4	15.50
32-fold diluted 10-min PAL	42.7	56.6 ± 1.9	5.44
5-min PAL with catalase	N.D.^c)^	9.2 ± 0.9	0.88
1.5-fold diluted 10-min PAL with catalase	N.D.^c)^	10.8 ± 1.3	1.04

^a)^Each of the solution has been adjusted by adding 20% (v/v) of 0.1 M citrate buffer (pH 6.1) in advance

^b)^Here the concentration of H_2_O_2_ in untreated 5-min and 10-min PAL were 1299, 1650 μM, respectively

^c)^Not detected

### Effects of PAL on the expression of γH2AX

**Fig. 6:**
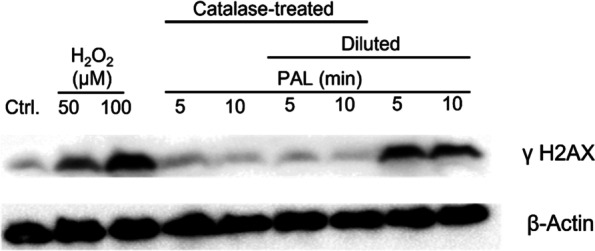
Western blot analysis of γH2AX in HeLa cells. Cells were exposed to pH-adjusted lactec, 50 μM or 100 μM H_2_O_2_ in HBS, or 5-min and 10-min PAL with / without dilution or catalase treatment. After western blotting, immunodetection was performed with an antibody specific to γH2AX. β-actin was used as a loading control

We analyzed the levels of phosphorylated histone H2AX (**γ**H2AX), which is related to DNA damage in cells. Hydrogen peroxide solution (1 M) was diluted into HBS (10 mM HEPES pH 7.4, 150 mM NaCl, 5 mM KCl, 2 mM CaCl_2_, 1 mM MgCl_2_, 0.1% glucose) at a final concentration of 50 μM or 100 μM. Citrate buffer (pH 6.1) was used for pH adjustment in PALs. Cells were treated with the HBS, PALs, catalase-treated or diluted solutions for 2 h and evaluated by western blot analysis for γH2AX expression (Fig. 6). The levels of γH2AX in H_2_O_2_-treated cells were higher than in the lactec-treated cells (control) in a dose-dependent manner. The levels of γH2AX in HeLa cells treated with catalase-treated, pH-adjusted PALs were similar to those in the control. These results indicate that H_2_O_2_ is primarily responsible for the accumulation of γH2AX in HeLa cells.

### Cell cycle analyses of PAL-treated HeLa cells

**Fig. 7:**
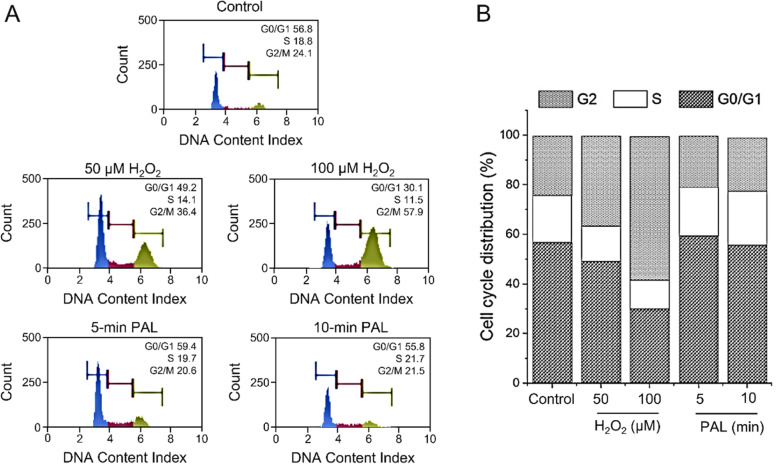
No effects of pH-adjusted, catalase-treated PALs on the cell cycle of HeLa cells. The flow cytometry (**A**) and the percent distribution of the treated cells in each cell cycle phase (**B**)

Genotoxic insults such as ionizing radiation and exposure to alkylating reagent induces G2 arrest of the cell cycle in HeLa cells [33]. We examined whether G2 arrest occurs in HeLa cells treated with pH-adjusted and catalase-treated PAL. Cells were treated with pH-adjusted lactec (control), 50 or 100 μM H_2_O_2_ in HBS, or catalase-treated 5-min or 10-min PAL. Flow cytometry showed that treatment with H_2_O_2_ increased the number of cells in the G2 phase and decreased those in the S phase (Fig. 7), indicating that H_2_O_2_ induces cell cycle arrest in the G2 phase. No G2 arrest was observed in cells subjected to catalase-treated 5-min or 10-min PAL. These results suggest that the active organic compound(s), in addition to H_2_O_2_, in the PALs does not induce G2 arrest of the cell cycle in HeLa cells.

## Discussion

The results presented here demonstrate that PAL has a killing effect on cancer cells, consistent with previous studies reporting the apoptosis of cancer cells treated with diluted PALs. Plasma irradiation generates reactive oxygen species (ROS) such as •OH, HO_2_ H_2_O_2_ in plasma plume. ROS can promote H_2_O_2_ generation and can dissolve in water [34–37], the likely main production pathway of H_2_O_2_ in aqueous solution A reduction in pH can be interpreted as the generation of •OH and H_2_O_2_ in aqueous solution and is dependent on the concentration of organic compounds (sodium lactate ≤28 mM) via •OH + H_2_O_2_ → H^+^ + •OO^—^ + H_2_O [34].

After 10 min irradiation with plasma, the pH value of PAL decreased to about 4.7, indicating that PAL is not suitable for direct application to living cells. Ringer’s lactate solution does not have a strong buffering action. We therefore first used phosphate buffer (pH 7.2) to evaluate the genotoxicity of PAL and observed no cell-killing activity by PAL pH 7.2 treated with catalase. Previous reports demonstrated that PALs diluted 16- or 32-fold with lactec with pH values of 5.9 and 6.0, respectively, shows a killing effect on cancer cells [26]. These observations led us to hypothesize that a compound other than H_2_O_2_ in PAL might be active around pH 6.0 but not at pH 7.2. Thus, we used citrate buffer instead of phosphate buffer to adjust the pH of PAL and found that catalase-treated PAL pH 5.9 killed HeLa cells. Around 40% of HeLa cells survived and formed colonies (Figs. 2, 4) after treatment with lactec pH 5.9, while 20% survived (Fig. 3) and 1.5% formed colonies (Fig. 4) after treatment with catalase-treated PAL pH 5.9. These results indicated the presence of one or more active compounds probably derived from lactate that are active at pH 5.9 but not at pH 7.2.

Catalase-treated PAL pH 5.9 killed HeLa cells but did not induce mutation at the *HPRT1* locus. Cells treated with diluted PAL containing 65.3 μM or 42.7 μM H_2_O_2_ resulted in a 15.5-fold or 5.4-fold increase in mutation frequency, respectively, compared to untreated cells. However, cells treated with catalase-treated PAL with a pH of 6.1 adjusted with citrate buffer showed almost the same level of mutation frequency compared with the control. These results indicate that PAL did not contain any compound aside from H_2_O_2_ that drastically increases the mutation frequency of the *HPRT1* gene in HeLa cells. In addition, we detected no apparent increase in γH2AX or cell cycle arrest in HeLa cells after treatment with pH-adjusted, catalase-treated PAL. The above results provide no strong evidence that PAL contains a genotoxic compound aside from H_2_O_2_. Thus, it is possible that PAL contains new compounds or species that might be ideal candidates as novel anticancer therapeutic reagents.

We previously detected specific compounds such as glyoxylate and 2,3-dimethyltartrate presented in plasma-activated L-sodium lactate solution that exhibited a cytotoxic effect specifically on cancer cells [26]. Thus, it is possible that 2,3-Dimethyltartrate is the active compound in PAL used in this study. In addition, we also showed that PAL specifically regulates cell death not by oxidative stress but by significant metabolomic changes [30]. However, the precise mechanisms underlying the antiproliferative effects of the active compound(s) in PAL remain unknown. Further studies are needed to identify the active compounds in PAL and to comprehensively understand the mechanisms of the specific anti-tumor effects of PAL.

## Conclusions

PAL (plasma activated lactec) is produced by the irradiation of Ringer’s lactate solution with NEAPP. PAL contains organic compounds derived from lactate, in addition to H^+^ and H_2_O_2_. Catalase-treated PAL pH 5.9 killed HeLa cells, whereas catalase-treated PAL pH 7.2 did not. Catalase-treated PAL pH 5.9 did not increase the mutation frequency of the *HPRT1* locus, γH2AX accumulation, or G2 arrest of the cell cycle in HeLa cells. Combining these results, PAL contains one or several non-genotoxic compounds or species active at pH 5.9 but inactive at pH 7.2 that kill HeLa cells. Thus, it is possible that the active compounds in PAL might be targeted as novel anticancer therapeutic drugs in the future.

## Data Availability

The datasets generated and/or analyzed during the current study are available from the corresponding authors upon reasonable request.
